# A Case of Prehospital Magnesium Sulfate Extravasation

**DOI:** 10.5811/cpcem.34874

**Published:** 2025-03-20

**Authors:** Sean Bilodeau, Michael Bohanske, Kate Zimmerman, Eric Wellman, Matthew Sholl

**Affiliations:** Tufts University School of Medicine, MaineHealth Maine Medical Center, Department of Emergency Medicine, Portland, Maine

**Keywords:** IV infiltration, extravasation, magnesium sulfate, prehospital IV access

## Abstract

**Case Presentation:**

A 73-year-old female with chronic obstructive pulmonary disease presented via emergency medical services (EMS) for shortness of breath. She was found to be hypoxic, tachypneic, and in notable distress by EMS. She was treated with inhaled albuterol, oral dexamethasone, and intravenous (IV) magnesium sulfate. Upon arrival to the emergency department her left hand was noted to have significant bleeding, and on further investigation it was determined that the IV catheter had inadvertently become dislodged causing medication extravasation, causing the magnesium to enter the subcutaneous space. The bleeding was significant and noted to be pulsatile; a tourniquet was applied; vascular damage was noted and was ultimately ligated by the trauma surgery service.

**Discussion:**

Intravenous medication administration is ubiquitous with emergency care in both the hospital and prehospital environments. Medication use is paramount to treatment of a vast majority of emergent clinical conditions; furthermore, the route of administration is often via IV in the patient with emergent illness. The placement of IV catheters is a skill that nurses, paramedics, and advanced emergency medical technicians learn early in their training. The care team is tasked with starting IV lines but also in monitoring them and ensuring medication is delivered into the systemic circulation and not elsewhere. Certain medications, notably potassium preparations and vasoactive medications, are known vesicants. We present a case of vascular extravasation of magnesium sulfate, not known for causing tissue damage, which led to significant vascular injury. This case highlights the need for prehospital professionals as well as members of the emergency department care team to be ever vigilant for medication extravasation.

## CASE PRESENTATION

**Image 1:**
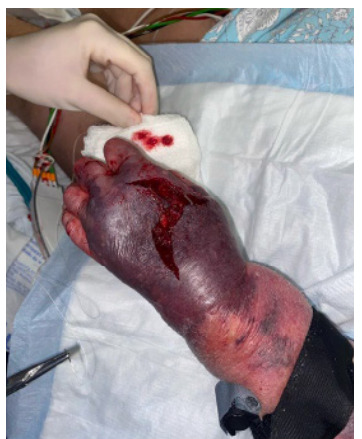
Dorsal surface of left hand with skin tear and ecchymosis status post extravasation.

**Image 2:**
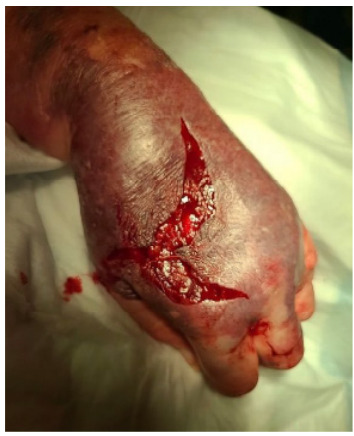
Dorsal surface of left hand status post vascular ligation.

A 73-year-old female with a history of chronic obstructive pulmonary disease (COPD) called emergency medical services (EMS) for worsening shortness of breath. The patient was found to be hypoxic despite home oxygen. Concern for COPD exacerbation prompted bronchodilators via nebulizer as well as oral dexamethasone. A 22-gauge intravenous (IV) catheter was placed with standard tactile technique in the dorsum of the left hand for administration of magnesium sulfate. There were two attempts at IV cannulation, and it was unclear why the dorsal hand was chosen as a site. Of note, no other medications or fluids had been administered through the IV catheter. In the emergency department the patient’s hand was edematous with concern for IV infiltration, and the IV was subsequently removed. Significant hemorrhage was noted with pulsatile bright red blood, a tourniquet was applied, and surgery was able to ligate the culprit vessel, which was found to be arterial (Images 1 and 2). During her hospital stay the wound improved, and she was discharged after resolution of a COPD exacerbation with outpatient, wound care follow-up.

## DISCUSSION

Intravenous catheter infiltration and medication extravasation are well-known complications of IV medication administration. However, extravasation causing vascular injury to point of significant hemorrhage has not been well described in the literature. The extravasation of magnesium sulfate has not been known to cause significant tissue injury; however, in this patient the magnesium sulfate was presumed to be the culprit, as there were no other medications administered through the IV, and the total infusion volume could not have been more than 104 milliliters (mL). Per local EMS protocols, magnesium is stocked in 1 gram (g)/2 mL vials and diluted in 100 mL of normal saline for a total dose of 2 g diluted in 104 mL of normal saline. Infusions are administered by infusion pump over 15 minutes.

**CPC-EM Capsule**

What do we already know about this clinical entity?

*Magnesium sulfate is not known to cause tissue injury when subject to extravasation*.

What is the major impact of the image(s)?

*Magnesium sulfate was the culprit for acute vascular injury when extravasated in this case; clinicians should be aware to mix and administer this medication appropriately*.

How might this improve emergency medicine practice?

*Monitoring for intravenous extravasation, even medications previously considered to be benign, is important for clinicians who administer medications via this route*.

The IV was placed in the ambulance while driving to the hospital. There are multiple potential issues with this case that could have led to the vascular and subcutaneous injury. The EMS report indicates that two IV attempts were made and that IV placement was confirmed with blood return; however, this could have certainly been in error. It is possible that the IV was inadvertently placed into an artery and then became dislodged leading to bleeding. The movement of the ambulance as well as the possibility of the successful IV being placed more distal in the same vein as the prior attempt could have led to extravasation as well. While magnesium is directed to be diluted and infused via infusion pump, it is possible that the medication was given undiluted via IV push. This concentrated form has the potential to be much more cytotoxic. Magnesium sulfate has not been known to be an active vesicant and has been considered relatively safe in the setting of inadvertent, soft-tissue exposure. However, if given in a concentrated form it would be more caustic. Taogoshi et al showed that higher concentrations of magnesium sulfate, greater than 8%, were cytotoxic in a rat model.^1^

Treatment for medication extravasation is dependent on the medication being used. In the case of magnesium sulfate, which does not have an antidote, the accepted treatment is to stop the infusion, withdraw as much medication out of the catheter with a syringe as possible, and then remove the catheter.^2^ Saline or other irrigation should not be infused into the catheter prior to removal. The affected extremity should then be elevated, bandaged, and a warm pack applied. The affected area should be checked at regular intervals for tension, bleeding, and vascular and neurologic changes.

This case demonstrates the need for heightened awareness when administering all medications, not just vesicants. This is of particular concern for EMS agencies placing IVs and monitoring them enroute. The anatomical location (dorsum of the hand) and non-ideal conditions (patient’s home and in a moving ambulance), as well as medication characteristics, all lead to significant injury.
